# Cerebral Sinus Thrombosis Secondary to SARS-CoV-2 Infection

**DOI:** 10.1155/2021/6640368

**Published:** 2021-03-03

**Authors:** Mojtaba Khazaei, Kiana Karimi, Parinaz Sedighi, Salman Khazaei

**Affiliations:** ^1^Department of Neurology, Hamadan University of Medical Sciences, Hamadan, Iran; ^2^Student Research Committee, Hamadan University of Medical Sciences, Hamadan, Iran; ^3^Universal Scientific Education and Research Network (USERN), Tehran, Iran; ^4^Research Center for Health Sciences, Hamadan University of Medical Sciences, Hamadan, Iran

## Abstract

Coronavirus disease-19 (COVID‐19) is a novel infectious disease and every day we are learning more about its various clinical features and complications. Different studies during the pandemic have shown various neurological manifestations secondary to the infection such as stroke due to cerebral vessel thrombosis. Herein, we presented a 57-year-old man admitted to our hospital with gradual headache, seizure, and decreasing level of consciousness. Three weeks earlier, he was diagnosed with COVID-19 and mild to moderate respiratory problems. Decreased level of consciousness made physicians intubate the patient and initiate mechanical ventilation in the intensive care unit (ICU). Treatment was initiated with phenytoin. Brain CT scan showed right transverse sinus and cortical vein thrombosis with subarachnoid hemorrhage. He received successful anticoagulant therapy, with further improvement in oxygenation, and discharged with a good general condition. This case is important because several neurological complications of COVID-19 should be noticed and managed by appropriate treatment according to the patient's condition.

## 1. Introduction

Stroke is the second leading cause of death and one of the most debilitating neurological diseases [1]. Cerebral venous thrombosis is considered an important cause of ischemic stroke. The prevalence of this thrombosis in Iran is about 12 per million people, which is much higher than in European and American countries [2]. This condition used to be considered rare, but now it is one of the most common brain diseases, especially in the Middle East [3]. Symptoms vary from mild to severe headaches to focal brain symptoms and loss of consciousness. The first step in the management of this condition is to use intravenous heparin and replace it with oral warfarin after a few days, based on stroke guidelines [4]. There are several known causes for cerebral sinus thrombosis, including pregnancy, dehydration, hereditary thrombophilia, history of trauma or surgery, using oral contraceptives, vasculitis, and ulcerative colitis. Since the end of 2019, with the spread of the severe acute respiratory syndrome coronavirus-2 (SARS-CoV-2) disease, concerns about arterial and venous thrombosis and also neurological complications have increased in this viral disease [5]. Neurological complications due to COVID-19 have increased which can be related to the hyperinflammatory and hypercoagulable states of viral infection. Central nervous system (CNS) disorders include encephalopathy, encephalitis, meningitis, ischemic and hemorrhagic stroke, and venous sinus thrombosis [6]. Different mechanisms have been suggested to explain thrombosis secondary to COVID-19. One of the explanations is the hypercoagulation state, due to rises in interleukin-6 (IL-6) and C reactive protein (CRP) levels [7]. Evidence of coagulation abnormalities has been observed in some patients with COVID-19, including thrombocytosis, hyperfibrinogenemia, elevated prothrombin time (PT) and partial thromboplastin time (PTT), elevated fibrin D-dimer, and elevated von Willebrand factor levels [8]. Cardiac and peripheral arteries, as well as cerebral arteries, are at risk for thrombosis. A way to prevent thrombosis and its complications is the use of anticoagulants as prophylaxis. It is estimated that about 6% of COVID-19 patients develop cerebrovascular complications. Also, in these patients, CT scan and brain image studies strongly confirm the association of COVID-19 with stroke [9, 10]. Here, we present a 57-year-old man presenting with headache and lowering level of consciousness with a history of COVID-19 three weeks before admission, who was diagnosed with cerebral venous thrombosis and underwent successful anticoagulation therapy.

## 2. Case Presentation

**Figure 1:**
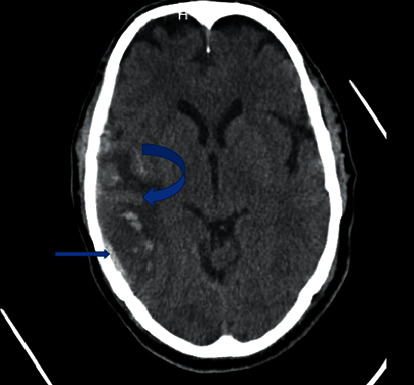
First brain CT scan showing cortical vein thrombosis (inferior anastomosis vein) (rectus arrow) with right temporal infarction in which transformation to hemorrhage occurs (curved arrow).

**Figure 2:**
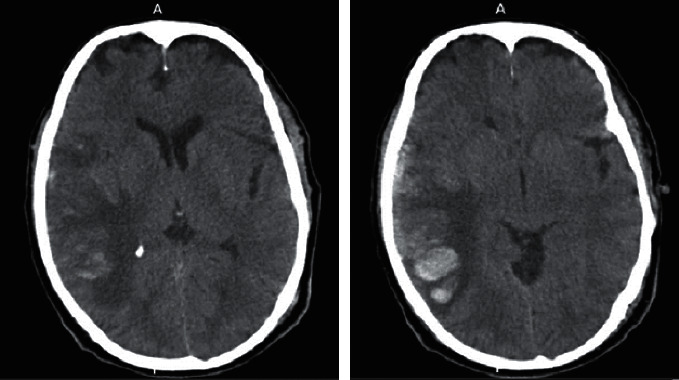
Second brain CT scan.

**Figure 3:**
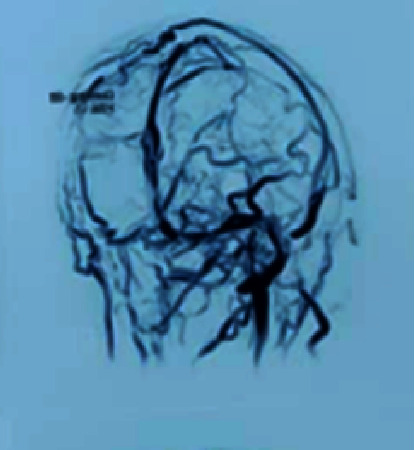
Brain MRV showing absence of right transverse sinus.

**Figure 4:**
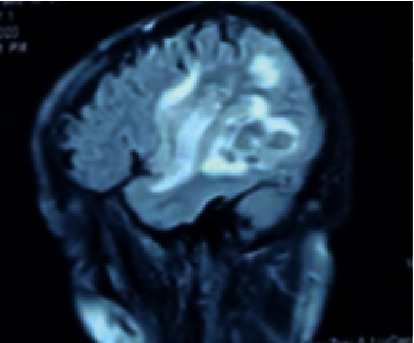
Brain MRI (FLAIR) in the sagittal plane showing hyperintensity in the right temporal lobe.

A 57-year-old man was admitted to the emergency department of our hospital. Two days before admission, he had headache, followed by left hemiplegia. On the next day, he experienced two episodes of seizure (generalized tonic-clonic) and each one continued for five minutes and he was not conscious between the two episodes. After the seizures, the patient's consciousness remained low. On admission, the patient was unconscious and agitated. His past medical history was unremarkable for any chronic diseases including diabetes mellitus, hypertension, and ischemic heart disease. The only considerable point was the SARS-CoV-2 infection three weeks ago and his symptoms were completely recovered. He did not have a history of head trauma. He did not smoke and did not use any kind of drugs before admission. On the first visit, the Glasgow Coma Scale (GCS) was two, vital signs including blood pressure, respiratory rate, heart rate, and temperature were within normal ranges, and O_2_ saturation was 87.5%. The pupils were mid-dilated and were not responsive to light. The doll's eye reflex was positive. Respiratory, abdominal, heart, and other physical examinations were unremarkable. The patient was admitted to the intensive care unit (ICU) and was intubated due to unconsciousness and agitation. Also, the first dosage of diazepam and sodium valproate was administered. Initial laboratory evaluations were performed, and the results are provided in Table 1. Midazolam, dexamethasone, phenytoin, sodium valproate, vancomycin, and meropenem were administered. On the second day of admission, the patient was auto-extubated due to agitation. O_2_ saturation was 96%, so after extubation, the patient was only supported by an O_2_ face mask. A brain CT scan was requested, and it revealed thrombosis in cortical veins and right transverse sinus in addition to subarachnoid hemorrhage (SAH) (Figure 1). D-Dimer test was done, and the result was 4654 ng/ml. After the diagnosis of thrombosis in brain veins, 7000 units of heparin were prescribed as a single dose, followed by infusion at 1000 units/kg, and heparin was monitored by the PTT. After achieving the therapeutic goal of heparin therapy, it was changed to warfarin after three days. The challenging point in the management of the patient was the variations in the INR (international normalized ratio) level that raised over four even with the administration of 1.25 mg of warfarin. Fortunately, there was no life-threatening bleeding. On the third day of admission, the patient's consciousness improved and the GCS was 13. The patient continued to be monitored in the ICU for five days. On the fifth day, he was transferred to the neurology ward and the second brain CT scan was requested for follow-up and it revealed parenchymal hemorrhage in the middle and posterior right temporal lobe inconsistent with the previous finding on the first scan (Figure 2). Also, magnetic resonance imaging (MRI) and magnetic resonance venography (MRV) were done and confirmed the findings of CT scan (Figures 3 and 4). Additional examinations for vasculitis and thrombophilia including antiphospholipid syndrome (APS) profile, C and S protein, and factor V Leiden were all negative. Finally, the patient was discharged with a good general condition, and mild dysarthria which was completely recovered on the follow-up visit. Warfarin was changed to apixaban after two weeks due to variation in the therapeutic range of warfarin, and the patient did not present any signs of thrombosis on the follow-up visits.

## 3. Discussion

The outbreak of the SARS-CoV-2 infection has become a serious challenge to all healthcare settings worldwide. Various central and peripheral neurologic complications have been reported among patients with different degrees of infection severity. It is estimated that about 30% of patients who were hospitalized due to COVID-19 develop neurological complications at some point in their treatment [11]. The spectrum of neurological symptoms in these patients includes stroke, sinus thrombosis, encephalomyelitis, encephalopathy, meningitis, ageusia and anosmia, muscle injury, and the Guillain–Barre syndrome [6]. COVID-19-related ischemic strokes are more common than hemorrhagic strokes [12]. Therefore, in any patient with symptoms of stroke, it is necessary to conduct workup for COVID-19 while we are in the pandemic. Strokes are mostly due to arterial involvement and less frequent by venous thrombosis [13]. Venous strokes can be caused by sinus or cortical vein thrombosis, and in this patient, both the sinus and the cortical veins were blocked. Presentation of venous involvement can vary from a simple headache to a deep loss of consciousness, in which seizures and loss of consciousness were the main symptoms in our patient [14].

Zayet et al. reported two cases of cerebral stroke with multiple infarctions secondary to COVID-19 in which patients were under anticoagulation therapy as prophylaxis for atrial fibrillation due to the previous history of cardiovascular disease. Both cases developed alterations in consciousness and one of them presented with hemiplegia, the same manifestations as our patient, but none of them had seizures. Finally, one of the patients died, despite supportive care and anticoagulation therapy. The cases demonstrate that COVID-19-induced hypercoagulation state can be severe as it can cause thrombosis in patients even under anticoagulation therapy [15]. Also, Fara et al. reported three cases of thrombosis and stroke in patients with mild SARS-CoV-2 infection in which all of them first presented with hemiplegia. One of the patients was under anticoagulation therapy (subtherapeutic dosage) due to a history of deep vein thrombosis (DVT) two months before. Finally, all of them were treated by proper anticoagulation therapy with complete resolution of thrombosis. The three patients had mild respiratory symptoms and positive polymerase chain reaction (PCR) tests inconsistent with CNS manifestations, but in our patient, cerebral hemorrhage developed three weeks later. Hence, we can conclude that COVID-19 can have both acute and delayed-type CNS complications [16].

A considerable point about this patient is clinicians should be careful about various types of CNS (central nervous system) involvement in patients with COVID-19 and they should treat the CNS complications besides the infection. And also, the treatment plan may differ from the treatment of the same problem in individuals without SARS-CoV-2 infection. The treatment plans for CNS involvement might be modified as we get more experienced during the pandemic.

## Figures and Tables

**Table 1:** Initial laboratory evaluations.

	Result	Unit
*Hematology tests*		
White blood cells (WBC)	15700	10^3^/*μ*l
Red blood cells (RBC)	5.62	10^6^/*μ*l
Hemoglobin (HB)	17.2	g/dl
Platelets (PLT)	200	10^3^/*μ*l
Erythrocyte sedimentation rate (ESR)	22	m/h

*Biochemistry tests*		
Blood sugar	229	mg/dl
Urea	35	mg/dl
Aspartate aminotransferase (AST)	53	mg/dl
Alanine aminotransferase (ALT)	95	mg/dl
Sodium	135	mEq/L
Potassium	4.9	mEq/L
Calcium	8.5	mg/dl
Phosphorus	2.8	mg/dl
Creatine phosphokinase (CPK)	381	U/L
Lactate dehydrogenase (LDH)	658	U/L
Creatine kinase-MB (CK-MB)	32	IU/L
Troponin	Negative	Qualitative
Immunoserology test	Result	Unit
C-reactive protein (CRP)	+++	Qualitative
Coagulation tests	Results	Unit
Prothrombin time (PT)	10	Seconds
International normalized ratio (INR)	1	—
Partial thromboplastin time (PTT)	24	Seconds

*Blood gas*		
pH	7.4	—
pCO_2_	42.1	mmHg
pO_2_	54	mmHg
HCO_3_^−^	26.1	mmol/L

*Urine analysis and culture*		
Color	Yellow	—
Appearance	Semiturbid	—
Specific gravity	1.015	—
pH	8	—
Proteins	Negative	—
Glucose	Positive	—
Ketones	Negative	—
Blood	Positive	—
Bilirubin	Negative	—
Urobilinogen	Negative	—
Bacteria	Few	—
Urine culture	No bacterial growth after 24 hours	—

## Data Availability

The data have been gathered from the patient's medical record with the patient's satisfaction (personal information of the patient including name is not mentioned in the text).
